# Bad science: International organizations and the indirect power of global benchmarking

**DOI:** 10.1177/1354066117719320

**Published:** 2017-07-31

**Authors:** André Broome, Alexandra Homolar, Matthias Kranke

**Affiliations:** University of Warwick, UK; University of Warwick, UK; University of Warwick, UK

**Keywords:** Business regulation, foreign direct investment, global benchmarking, global governance, indirect power, international organizations

## Abstract

The production of transnational knowledge that is widely recognized as legitimate is a major source of influence for international organizations. To reinforce their expert status, international organizations increasingly produce global benchmarks that measure national performance across a range of issue areas. This article illustrates how international organization benchmarking is a significant source of indirect power in world politics by examining two prominent cases in which international organizations seek to shape the world through comparative metrics: (1) the World Bank–International Finance Corporation Ease of Doing Business ranking; and (2) the Organisation for Economic Co-operation and Development FDI Regulatory Restrictiveness Index. We argue that the legitimacy attached to these benchmarks because of the expertise of the international organizations that produce them is highly problematic for two reasons. First, both benchmarks oversimplify the evaluation of relative national performance, misrepresenting contested political values drawn from a specific transnational paradigm as empirical facts. Second, they entrench an arbitrary division in the international arena between ‘ideal’ and ‘pathological’ types of national performance, which (re)produces social hierarchies among states. We argue that the ways in which international organizations use benchmarking to orient how political actors understand best practices, advocate policy changes and attribute political responsibility thus constitutes ‘bad science’. Extending research on processes of paradigm maintenance and the influence of international organizations as teachers of norms or judges of norm compliance, we show how the indirect power that international organizations exercise as *evaluators* of relative national performance through benchmarking can be highly consequential for the definition of states’ policy priorities.

## Introduction

Since the turn of the century, there has been an unprecedented expansion in the use of benchmarking as a governing strategy across all areas of social and economic life in a growing number of countries. At the same time as benchmarking has become a core tool of domestic regulation, transnational actors have increasingly produced ratings and rankings to assess relative national performance at the global level. Benchmarks have become integral to the comparative evaluation of countries’ institutional design, policy agendas and behaviour across issue areas as diverse as global development goals (Clegg, 2015), climate change action (Kuzemko, 2015), corruption (Baumann, 2017), human security (Homolar, 2015), international human rights norms (Harrison and Sekalala, 2015), national economic policies and institutions (Sending and Lie, 2015), political freedom (Bush, forthcoming), and poverty reduction (Freistein, 2016). Global benchmarks based on country rankings are deceptively easy to communicate and consume around the world.

Consistent with the rise of benchmarking, International Relations scholars have recently begun to direct attention to the politics of utilizing comparative national performance metrics (Hansen and Mühlen-Schulte, 2012; Mügge, 2016). Within this emerging research agenda, work has focused on how — and to what extent — benchmarks directly influence the behaviour of target states (Cooley and Snyder, 2015), as well as how benchmarking practices draw on and reinforce existing transnational policy paradigms and dominant discourse (Broome and Quirk, 2015a; Fougner, 2008). This article contributes to such research by providing a conceptual critique of the production and dissemination of comparative national performance metrics by prominent international organizations (IOs). We connect the global governance role that IOs play in measuring and evaluating relative national performance to two strands of earlier research on how IOs seek to shape standards of ‘best practice’: (1) work on IOs as teachers of norms (Finnemore, 1993; Jacoby, 2001); and (2) work on IOs as judges of norm compliance (Hafner-Burton, 2008; Sharman, 2009). IOs were pioneers in developing benchmarks in the early 1990s, and of the more than 200 new global benchmarks that were created from 2000 to 2015, at least 40 were established by IOs (Global Benchmarking Database, 2017). Our emphasis on IOs as *evaluators* is in contrast to existing works in International Relations, which analyse benchmarking practices either as a general trend among different categories of transnational actors (Cooley and Snyder, 2015; see also Broome and Quirk, 2015b; Merry et al., 2015), or focus on a single benchmark that has been retrospectively identified as influencing state behaviour (Kelley and Simmons, 2015).

In this article, we explore how comparative performance metrics produced by IOs continue to be plagued by two problems that create and reproduce distorted images of the world. First, input factors differ far more than the methodologies used in the design of global benchmarks imply. Countries have vastly divergent structural positions (in both political and economic terms) and are endowed with sharply uneven capacities to implement domestic policies or to exercise international agency over global rules and policy norms. These differences tend to be glossed over in the process of operationalizing key concepts of national performance. Second, outputs often differ far less than the league tables portrayed in IO benchmarks indicate. Especially when they are produced in the form of rankings, benchmarks commonly exaggerate differences in performance between countries, obscuring a high degree of similarity in institutional forms and performance outcomes within country clusters (Høyland et al., 2012).

As these related input and output problems tend to be swept under the carpet by the political agents that produce and utilize them, IO benchmarks receive more scientific credibility than they deserve. We argue in this article that IO benchmarking is often troubled by questionable methodology and data collection biases because the use of transnational knowledge by IOs to produce global benchmarks cannot be separated from political values and policy reform preferences. Consequently, rather than underwriting the role they claim as ‘truth-tellers’ to their member states, through benchmarking, IOs help to maintain existing transnational policy paradigms and to legitimate existing hierarchies in world politics. Power relations are thus an inherent feature of the ‘bad science’ of IO benchmarking exercises. As the article demonstrates, benchmarks are a key source of *indirect power* for IOs to shape world politics according to their image of best practice in a given issue area, connecting their organizational expertise to the identification of ‘ideal’ and ‘pathological’ models of state policy and performance.

The article proceeds as follows. First, we build on the existing literature that emphasizes the workings of indirect power in order to establish the importance of understanding the role that IOs play as evaluators in world politics. We then expand on the conceptualization of the effects of IO benchmarking through a discussion of two empirical cases of IO benchmarking: (1) the World Bank–International Finance Corporation (IFC) Ease of Doing Business (EDB) ranking^1^; and (2) the Organisation for Economic Co-operation and Development (OECD) FDI Regulatory Restrictiveness Index (FDI Index). In each example, we show how the benchmark does not deserve its reputation as an aggregation of neutral observations of national progress in a given issue area. Instead, both suffer from construct and content validity problems because they lack a scientific basis for how they operationalize highly political concepts into what are perceived as objective, value-neutral categories. In the final section, we discuss how IO benchmarking functions as a mechanism of paradigm maintenance by promoting oversimplified images of the world as split between ideal and pathological forms of state policy and performance. Despite containing significant methodological problems and representing contested policy ideas as best practices, IO benchmarks achieve legitimacy — and thereby policy traction — by piggybacking on the status of the organizations that produce them as expert evaluators.

## IOs as evaluators of national performance

IOs wield power over other political actors both directly and indirectly. Until recently, most International Relations scholarship on how IOs shape world politics has focused on identifying and explaining how they are able to exercise direct power over states, primarily through carrot-and-stick approaches and the resolution of information problems. Within this literature, IOs are seen as influential if they are institutionally mandated and endowed with sufficient resources to organize bailouts for distressed economies, to coordinate crisis management policies or development financing, and to impose loan conditionality (Lütz and Kranke, 2014; Moschella, 2016; Nelson, 2014; Park and Strand, 2016). Research has also shown that IOs can exercise direct power over states by monitoring and enforcing compliance with international agreements (Simmons, 2000), as well as by directing and sponsoring capacity-building programmes that encompass international policy training and technical assistance initiatives (Broome and Seabrooke, 2015). Related work locates additional sources of direct IO influence in their mandated surveillance on behalf of member states (Lombardi and Woods, 2008), policy reform advice to national elites (Fang and Stone, 2012), and external approval of key policy settings (Hinterleitner et al., 2016).

**Figure 1.**
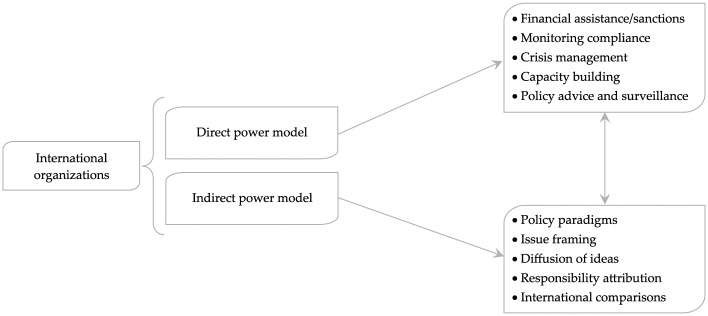
Direct and indirect expressions of IO power.

Channels of institutional influence in global governance stretch far beyond the visible sets of formal relations between transnational actors and national policymakers. International Relations scholarship has begun to pay greater attention to the ability of political agents to exercise ‘indirect control over the conditions of action of socially distant others’ (Barnett and Duvall, 2005: 48), even though these dynamics are often harder to observe and specify than more direct forms of coercion (see Figure 1). In our investigation of how IOs shape policy agendas and preferences through benchmarking, we directly speak to such scholarship, adding to two key research strands on the indirect power of IOs in world politics.

The first body of scholarship focuses on transnational socialization processes and provides insights into how IOs can serve as *teachers* of norms to states. This approach establishes IOs as institutional actors who are not only able to socialize policy elites into a common framework of understanding in a given issue area (Finnemore, 1993; Jacoby, 2001), but also foster ‘diagnostic coordination’ in how policy problems are defined and acted upon as policymakers move along ‘policy curves’ (Broome and Seabrooke, 2015). The second body of scholarship centres on the role of stigma, showing that some IOs function as *judges* that impose reputational costs on countries that fail to comply with international norms through stigmatization. Such forms of ‘shaming’ can occur through the blacklisting of states deemed to have violated particular normative standards, which both imposes a reputational sanction and triggers incentives for compliance (Hafner-Burton, 2008; Sharman, 2009). The explicit use or implicit threat of shaming provides IOs with a valuable tool for enforcing international norms, especially if stigmatization produces knock-on effects for aid flows, foreign investment or sovereign credit ratings (Chwieroth, 2015). We build on and extend these literatures on the indirect power of IOs by focusing on how IOs serve as *evaluators* of national performance via global benchmarking. As with socialization and stigmatization processes, the evaluative role that IOs play as benchmarkers can also serve as a mechanism of ‘paradigm maintenance’ (Wade, 1996) by delineating what does and does not count as legitimate transnational knowledge.

The notion that IOs exercise agential power through knowledge practices is consistent with existing constructivist literature in International Relations. Such scholarship has shown that their expert status and ‘cognitive authority’ helps IOs: to turn policy developments into governable issues, diffuse political ideas and set policy agendas (Broome and Seabrooke, 2012; Clegg, 2010; Hülsse, 2007); to validate and promote emergent norms (Park and Vetterlein, 2010; Weaver, 2008); and to develop, maintain and adapt transnational policy paradigms (Babb, 2013; Ban, 2016; Broad, 2006; Wade, 1996).

Indirect power frequently works through disciplinary dynamics that are far subtler than the ‘sticks’ of threats, sanctions or delegation. It is conceptualized here as relations that are rooted in the formation and control of what bodies of knowledge become accepted, or what counts, in Pierre Bourdieu’s (1989: 20) words, as ‘the legitimate vision of the world’. Based on this specific understanding of indirect power, we argue that IO benchmarks create meaning through knowledge-based articulations of the problems facing national officials and through the designation of countries’ standing relative to their peers, which establishes social hierarchies of ‘good’ and ‘bad’ performers. Such hierarchies connect the promotion of international norms with social pressure on states to conform to these standards of behaviour and policy design (Towns and Rumelili, 2017). We refer to the best practice model that a global comparative performance metric produces as an ‘image’: ‘Like a photographic image, it foregrounds a particular aspect of the world and excludes others’ (Fry and O’Hagan, 2000: 5; see also Hansen, 2011).

The country evaluations produced by IOs can be expected to carry greater weight than those produced by more openly partisan actors or cause-oriented organizations. The expert status that prominent IOs have achieved through their mandate and track record — backed by institutional resources in terms of expert human capital and combined with an organizational culture that privileges accepted positivist methodologies in knowledge production — enhances their influence in transnational policy debates. As a rich scholarly literature has already demonstrated, the most prominent IOs often seek to cultivate their standing among their respective audiences as ‘truth-tellers’ that can cut through the logjam of national interests, partisan differences and domestic veto-players by appealing to the rational-scientific basis of their policy knowledge and advice (Barnett and Finnemore, 2004; Béland and Orenstein, 2013; Broad, 2006; Broome and Seabrooke, 2012; Ecker-Ehrhardt, 2012; Kramarz and Momani, 2013). Recent case-study research suggests that IOs are perceived by citizens as having greater credibility than national governments in reporting on relative national performance, even when they are providing the same information (James and Petersen, 2017).

The expert authority with which IOs construct ratings and rankings of national performance therefore differs from other producers of global benchmarks, such as civil society organizations, market actors and states. Unlike activist organizations, IOs enjoy greater resources to exercise political leverage and benefit from an official mandate for action in a specified policy domain. Unlike market actors, IOs use benchmarks not for commercial gain, but to shape policy conversations and political agendas. Finally, unlike states, IOs do not pursue national interests by using benchmarks as a tool of foreign policy, which links indirect power to direct coercion. In contrast to these other types of benchmark producers, IOs rely more heavily on the recognition of their role as expert producers of transnational knowledge to gain public attention and policy traction.

## IO benchmarking in practice

IO benchmarks often enjoy a reputation as science-based metrics of comparative national performance that are removed from the political contests characteristic of many transnational processes. Despite the high degree of credibility assigned to them, as we will show, IO benchmarks suffer from construct and content validity problems that create distorted representations of the quality of country performance. Construct validity refers to whether the evaluation techniques used to construct a global benchmark effectively measure what they purport to measure, in particular: how effectively key concepts are defined, operationalized and weighted as a set of variables; whether consistent and high-quality cross-national data are available; and whether the various types of source material used are equally robust and internally coherent, which is especially challenging in the case of composite benchmarks. In short, construct validity concerns the degree of ontological fit between measurement techniques and empirical objectives. Content validity refers to whether the scope of a benchmark provides adequate coverage of the multiple dimensions of an issue area to effectively capture and measure performance, or whether critical dimensions are excluded (Michener, 2015: 187, 189).

In this section, we provide empirical illustrations of how IOs rely upon their expert status to disseminate seemingly neutral observations of national progress in a given issue area. Specifically, we discuss two benchmarks each of which is produced by a well-resourced IO that enjoys high prestige as a creator and disseminator of transnational policy knowledge: the EDB ranking and the FDI Index. We selected these two benchmarks from the Global Benchmarking Database (2017), an online repository of 275 global benchmarks that we created and continue to expand.^2^ Our rationale was to select benchmarks that differ in their core attributes, instead of choosing benchmarks from the same issue area or those produced by different categories of actors, as is common in recent comparative studies (see Michener, 2015). The cases differ along the following lines: what issue area they cover; whether they evaluate statutory regulations or performance outcomes; whether they translate the results into ratings (scores) or country rankings; what type of source material they use; how many countries they cover; and how much media attention they receive (see Table 1). The focus on IO benchmarks with different core attributes not only draws attention to the methodological limitations of benchmarks in a specific issue area, but also highlights the common factors at play in how different IOs use benchmarks as instruments of indirect power.

**Table 1.** Selected benchmark characteristics.

	EDB ranking	FDI Index
*Producer*	World Bank–International Finance Corporation	Organisation for Economic Co-operation and Development
*Issue area*	Business environment	Investment regulation
*Evaluation target*	Statutory regulations	Statutory regulations
*Results format*	Country rankings	Country ratings (scores)
*Source material*	Large-N expert survey	Review of statutory regulations
*Coverage*	190 countries	62 countries
*Media attention*	High	Low

### The World Bank–IFC EDB ranking

Few IO benchmarks have gained as much attention and generated as much controversy as the EDB ranking of the local business environment for small- and medium-sized private sector firms in World Bank member states. The benchmark has been published annually since 2006 as a core component of the flagship World Bank–IFC *Doing Business* report. The explicit foundation of the *Doing Business* report is the assumption that economic activity benefits from ‘Rules that set out and clarify property rights and facilitate the resolution of disputes … [a]nd ... that enhance the predictability of economic interactions’ (World Bank, 2016: 13). The report traces reform trajectories around the world, showcasing the ‘big strides in business regulation’ achieved ‘in every region’ and praising the ‘most improved’ countries (World Bank, 2015: 16, 35–53). Countries with a higher (= better) year-on-year aggregate score are highlighted with an upward arrow in the ranking, while the rankings are accompanied by short country reform summaries, which can additionally signal what each country has done well or badly since the previous ranking with a tick or a cross, respectively. However, positions at the top and bottom of the rankings tend to remain stable over time, as Table 2 illustrates.

**Table 2.** Top 10 and bottom 10 listings in the EDB ranking, 2015–2017.

	2015	2016	2017
*Top 10 countries*	Singapore (1)	Singapore (1)	New Zealand (1)
	New Zealand (2)	New Zealand (2)	Singapore (2)
	Hong Kong SAR, China (3)	Denmark (3)	Denmark (3)
	Denmark (4)	Korea, Rep. (4)	Hong Kong SAR, China (4)
	Korea, Rep. (5)	Hong Kong SAR, China (5)	Korea, Rep. (5)
	Norway (6)	United Kingdom (6)	Norway (6)
	United States (7)	United States (7)	United Kingdom (7)
	United Kingdom (8)	Sweden (8)	United States (8)
	Finland (9)	Norway (9)	Sweden (9)
	Australia (10)	Finland (10)	Macedonia, FYR (10)
*Bottom 10 countries*	Haiti (180)	Equatorial Guinea (180)	Haiti (181)
	Angola (181)	Angola (181)	Angola (182)
	Venezuela, RB (182)	Haiti (182)	Afghanistan (183)
	Afghanistan (183)	Chad (183)	Congo, Dem. Rep. (184)
	Congo, Dem. Rep. (184)	Congo, Dem. Rep. (184)	Central African Rep. (185)
	Chad (185)	Central African Rep. (185)	South Sudan (186)
	South Sudan (186)	Venezuela, RB (186)	Venezuela, RB (187)
	Central African Rep. (187)	South Sudan (187)	Libya (188)
	Libya (188)	Libya (188)	Eritrea (189)
	Eritrea (189)	Eritrea (189)	Somalia (190)

*Source*: Data retrieved from *Doing Business* reports, available at: http://www.doingbusiness.org/Reports

The EDB ranking encompasses 10 categories of economic governance that are used to measure the quality of the regulatory environment in each economy, focusing on the regulation of small- and medium-sized enterprises. The data are based on survey questionnaire responses from over 12,500 legal experts and business consultants in the 190 countries included in the report. The 10 categories of business regulation assessed to produce the ranking are: (1) ‘starting a business’; (2) ‘dealing with construction permits’; (3) ‘getting electricity’; (4) ‘registering property’; (5) ‘getting credit’; (6) ‘protecting minority investors’; (7) ‘paying taxes’; (8) ‘trading across borders’; (9) ‘enforcing contracts’; and (10) ‘resolving insolvency’. The *Doing Business* report also includes indicators on labour market regulation, but these have been excluded from the EDB ranking since 2011 following criticism from the International Labour Organization, the International Trade Union Confederation and other bodies of the validity of the ‘employing workers’ indicator and the causal assumptions that underpinned it (Lee et al., 2008). Reflecting the free market principle that labour markets work best with minimal protections for workers, in EDB rankings prior to *Doing Business 2008*, the ‘employing workers’ indicator ranked countries more positively based on how easy it was to dismiss workers, while restrictions on night work, such as overtime pay and scheduling of work hours limitations, were classified as ‘rigidities’, which received more negative scores (World Bank, 2017a). Index scores for each of the 10 individual components of the benchmark are produced through expert assessments of the legal context and procedures in each country, after these aspects have been analysed by World Bank–IFC officials and checked against statutory rules and regulations.

The EDB ranking is widely cited as ‘an authoritative and credible outside judge’ of a country’s business environment (Cooley, 2015: 1) and receives substantial press coverage and global media attention (Davis et al., 2012: 93). According to citation data provided by the World Bank (2016: 24, fn. 11), from the first *Doing Business* volume in 2003 to 2016, the report’s indicators have been dealt with in 2,182 peer-reviewed articles in academic journals and 6,296 online working papers. In addition to the high level of interest from journalists and academics alike, political leaders have made improving their countries’ EDB ranking an official objective of government policy (Besley, 2015: 99–100).

Consider the case of Russia’s commitment to structural economic reform, which formed part of a larger effort by the country’s government to attract higher rates of foreign investment. President Vladimir Putin explicitly referenced the EDB rankings to signal Russia’s move towards internationally recognized standards of ‘doing business’. In special decrees published in May 2012, Putin announced an interim goal for Russia to reach 50th place in the EDB ranking by 2015, and a longer-term target of achieving 20th position by 2018 (Pomeranz and Smith, 2016). Putin’s interim goal was only missed by the narrowest of margins: the country’s position improved rapidly from its EDB ranking of 120th in 2012 to 112th in 2013, 92nd in 2014, 62nd in 2015, 51st in 2016 and 40th in 2017. This substantial rise in Russia’s EDB ranking was made possible by selective liberalizing reforms, through which the Russian government targeted the specific areas covered by the indicators used to produce the EDB ranking, specifically policy changes relating to ‘starting a business’, ‘getting electricity’, ‘registering property’, ‘getting credit’ and ‘paying taxes’ (World Bank, 2015: 179).

The value attached to the benchmark when it comes to the investment promotion strategy adopted by Russia’s president, as well as leaders from countries as diverse as India (Besley, 2015) and the Republic of Georgia (Schueth, 2015), suggests that it has become a symbolic marker of national status and relative economic competitiveness. As then-World Bank Chief Economist Kaushik Basu claimed, the EDB has achieved the status of ‘one of the world’s most influential policy publications’ (World Bank, 2015: iv) and thus carries a significant degree of expert legitimacy. In turn, the knowledge-based authority associated with the benchmark implies that there is a risk that IOs and other agencies start ‘teaching to the test’ to obtain policy adjustments only in the specific areas covered by the EDB indicators. The EDB ranking thus constrains the space for tailoring reform proposals to individual countries’ needs: it places a penalty on fostering the development of innovative policy alternatives that lie outside the ‘EDB box’ and that do not reflect the neoliberal policy paradigm at the heart of the conceptual categories used to construct the ranking.

For the World Bank and the IFC, the EDB ranking provides a powerful symbolic instrument that establishes a hierarchy of regulatory shortcomings across countries, as well as shaping policy conversations and guiding officials towards what are presented as optimal policy reforms. In a 2008 official evaluation of the quality and effects of the indicators used to produce the EDB ranking by the Independent Evaluation Group (2008: 43), the World Bank Group’s semi-independent watchdog, staff maintained that ‘Ranking with peers provides incentives for reforms, not the survey itself’. While the benchmark ‘scores economies based on how business friendly their regulatory systems are’ (World Bank, 2016: 5), there is significant scope for ‘inadequate’ regulation (based on a limited number of restrictions) to be conflated with more ‘efficient’ regulation (Independent Evaluation Group, 2008: 32). The image of the economy articulated in the *Doing Business* report thus represents regulation as a burden on business and a constraint on economic growth, which should be reduced to a minimum. This naturalization of a particular conception of a liberal market economy as an organizational ideal marginalizes considerations of alternative policy practices and goals via a narrow focus on regulatory design for the purpose of enabling business freedom.

Since the 2015 edition, the EDB ranking has been obtained through the calculation of a novel ‘distance to frontier’ score, which aims to benchmark countries against regulatory ‘best practice’. The score expresses the difference in performance for each country compared with the best performance recorded by any country in each of the 10 sets of *Doing Business* indicators since 2005, or the third year in which data have been collected for indicators introduced after 2005. Distance to frontier scores range from 0 (the ‘worst performance’) to 100 (‘the frontier’, or best recorded performance). The 10 distance to frontier scores for each set of indicators are aggregated into a simple average to produce an overall distance to frontier score for each country (World Bank, 2016: 167).

While the World Bank (2016: 164) claims that ‘The distance to frontier score captures the gap between an economy’s performance and a measure of best practice’, it analytically privileges fewer and cheaper restrictions as ideal ‘best practice’. For example, New Zealand ‘set the frontier’ for two of the four ‘starting a business’ indicators, with an overall distance to frontier score in 2017 of 99.96. New Zealand requires only one procedure for starting a business, which can be completed in half a day at low cost (calculated at 0.3% of income per capita with no minimum requirement for paid-in capital) (World Bank, 2016: 165, 228). The country that scored worst on this indicator in 2017 is the Central African Republic, with a distance to frontier score of 31.36. The Central African Republic requires 10 procedures for starting a business, taking 22 days to complete at significant cost (calculated at 209.4% of income per capita, with a minimum paid-in capital requirement of 556.6% of income per capita) (World Bank, 2016: 198).

**Figure 2.**
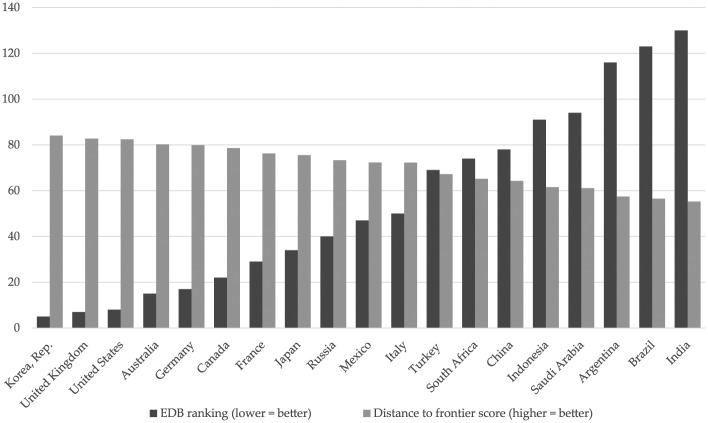
*Doing Business 2017* aggregate scores/rankings for G20 countries. *Source*: World Bank (2016).

The calculation of distance to frontier scores is underpinned by the neoliberal assumption that less intrusive regulations and lighter compliance costs for business are an effective and legitimate measure of economic efficiency. This serves as a form of paradigm maintenance by determining the yardstick with which national policy performance is evaluated. Distance to frontier scores also help to reveal how ordinal rankings can distort images of national performance. Figure 2 shows how the overall distance to frontier scores for many Group of Twenty (G20) countries are closely clustered. The 10 ‘best’ G20 countries received distance to frontier scores in *Doing Business 2017* that ranged from 84.07–72.29, indicating that the G20 country in 10th position was less than 12 percentage points behind the top G20 country in terms of the ‘regulatory frontier’ parameter. Once these same scores are converted into an ordinal ranking, however, the results imply a greater degree of variation across countries, ranging from 5th place (Korea) to 47th (Mexico).

The image of the world articulated in the EDB ranking reproduces contested ideals underlying World Bank assessments in country operations as value-neutral measurements of business regulations when it comes to assigning political responsibility. These evaluations assume: that national policies, not international structures, determine the quality of local business environments; that ‘excessive’ regulation of business impairs growth; and that international economic integration is beneficial for domestic economic performance in all circumstances. The EDB ranking likewise advocates domestic deregulation as a panacea and locates the need for political change solely at the domestic level, where stubborn policymakers and slow bureaucrats are construed as obstructing the long-overdue move towards economic liberalization. The EDB ranking is thus used as a means to assert the authority of the World Bank Group over economic and development policy challenges within a neoliberal policy paradigm, while constraining the scope for debating, designing and defending alternative national policy changes.

Nevertheless, the EDB ranking has not been universally accepted as a legitimate expert assessment of countries’ local business environment. Scholars have identified a range of conceptual omissions and methodological flaws in the EDB ranking, especially relating to the contentious issue of labour market regulation (Benjamin et al., 2010: 79–87; Berg and Cazes, 2008; Lee et al., 2008: 420–426). The EDB ranking has also received substantial criticism from a global coalition of civil society organizations, which saw the benchmarking exercise as undermining the World Bank’s broader credibility (Stichelmans, 2014). In light of these criticisms, two official reviews of the *Doing Business* report have lent support to proposals that the EDB ranking either needs to be significantly reformed (Independent Evaluation Group, 2008: 54) or perhaps even abandoned entirely (Independent *Doing Business* Report Review Panel, 2013: 4).

Due to these significant conceptual and methodological weaknesses, the EDB ranking conveys ‘misleading policy messages that invite simplistic and potentially erroneous policy conclusions’ (Berg and Cazes, 2008: 350). In addition to its methodological limitations, the EDB ranking serves as a source of rhetorical legitimation for a neoliberal policy paradigm, based on the evaluation of ‘good’ and ‘bad’ models of national performance using measurements and indicators that privilege free market principles. From the World Bank’s perspective, however, the benchmark helps to promote the organization’s broader aim of encouraging countries to more closely mirror the image of the world represented in the EDB ranking. This image of business freedom promotes a particularistic conception of a ‘good’ economy as one characterized by minimal public grip on the actions of private businesses. Like other development indicators produced by the World Bank, such as the international poverty line measure and income per capita country categories, the EDB ranking defines the identities of countries by means of ‘a hierarchical order in which some of them are placed above the others’ (Uribe, 2015: 138).

The EDB ranking thus presents and promotes a very particular image of the world, one in which political debates about regulatory reform are restricted to determining the operational priorities of the supply side of national economies. What business freedom from regulatory burdens means for different groups of workers and consumers, and the wider political ramifications that emerge from different ways of governing business activities, is precluded. The distributional effects and political consequences of restrictive or lax regulatory standards are ultimately irrelevant to a standard focal point for institutional and policy reforms: reduction of regulatory ‘red tape’ and economic liberalization in line with an ideal that endorses competition as the primary or sole means to generate economic and social progress (see Elias, 2013). Although business regulations may be used to shield a weak economy from external volatility, to constrain predatory business practices or to pursue broader developmental goals, these possibilities are not accounted for.

### The OECD FDI Index

Many IO benchmarks aim primarily at capturing the attention of elite actors in specialist policymaking fields, and seldom make front-page headlines or generate widespread civil society attention. An ideal-typical IO benchmark that targets a politically salient issue area but gains relatively little publicity is the OECD’s FDI Index, which aims to measure how restrictive countries’ statutory rules are on foreign direct investment (FDI). The FDI Index was established in 2003 to comparatively measure the restrictiveness of FDI rules on an occasional basis, and is jointly produced by the OECD Investment Division and the OECD Economics Department. Initially created for the years 1997, 2003 and 2006, it has been released annually since 2010. The number of countries assessed by the FDI Index has gradually expanded from 44 to 62. In its current format, it covers all G20 countries and current OECD members, as well as non-OECD adherents to the OECD’s Declaration on International Investment and Multinational Enterprises.

The 2003 version of the FDI Index measured ‘restrictiveness’ across nine sectors, calculating country scores based on a weighted average for sector scores from FDI and trade flow data (Golub, 2003: 93, 113). A modified 2006 version with adjusted industry scores measured essentially the same nine sectors weighted by the sectoral composition of overall inward FDI and trade flows for OECD countries (Koyama and Golub, 2006: 14). While the earlier versions of the FDI Index covered four secondary and five tertiary economic sectors, the 2010 revision of the classificatory system expanded its scope to incorporate four primary, seven secondary and 10 tertiary economic sectors plus real estate. These 22 equally weighted sectors are split into four dimensions of restrictions on FDI: (1) ‘foreign equity limitations’; (2) ‘screening or prior approval mechanisms’; (3) ‘restrictions on the employment of foreigners as key personnel’; and (4) ‘operational restrictions’, such as restrictions on landownership and the repatriation of capital. The restrictiveness of countries’ policy measures are assessed using indicators linked to the standards enshrined in the OECD Code of Liberalisation of Capital Movements (Kalinova et al., 2010: 6).

Based on an assessment of policy measures included within these four dimensions, a country receives a score with three decimal places on a scale from 0 (= ‘open’) to 1 (= ‘closed’) for each of the 22 economic sectors. To create an overall rating, the individual scores are translated into a simple average across sectors, with each sector equally weighted. The shift away from using sector scores weighted by FDI and trade flows towards a simple average removed the relationship between the individual sectors and global economic dynamics. This seemingly minor change in methodology in 2010 had a significant impact upon the scores for some countries, especially those with large primary industry sectors. For example, New Zealand saw its overall score worsen from 0.170 in 2006 (when it was rated as the 28th most open to FDI) to 0.263 in 2010 (the 42nd most open to FDI), largely as a consequence of foreign equity restrictions in fisheries and other restrictions in primary industries in the country.

Unlike many IO benchmarks, the FDI Index does not produce a formal ranking of countries’ scores, but merely presents countries’ aggregate ratings in alphabetical order. The FDI Index therefore avoids the methodological problem of artificially inflating the differences between countries’ positions through visual representations of comparative performance, such as league tables and heat maps (Broome and Quirk, 2017), which plague many other ordinal rankings of national performance. Nevertheless, the country ratings assigned in the FDI Index establish a clear hierarchy when it comes to investment restrictions among the countries, which centres on representing fewer policy restrictions on foreign investment as ideal and promoting the objective of increasing the process of liberalization over time. Moreover, the scores can be manually translated into a ranking with ease. Table 3 shows that, of the two IO benchmarks examined here, the OECD’s FDI Index exhibits a greater degree of stability in the top and the bottom 10 positions over time.

**Table 3.** Top 10 and bottom 10 listings in the FDI Regulatory Restrictiveness Index, 2014–2016.

	2014	2015	2016
*Top 10 countries*	Luxembourg (1)	Luxembourg (1)	Luxembourg (1)
	Portugal (2)	Portugal (2)	Portugal (2)
	Slovenia (2)	Slovenia (2)	Slovenia (2)
	Romania (4)	Romania (4)	Romania (4)
	Czech Republic (5)	Czech Republic (5)	Czech Republic (5)
	Netherlands (6)	Netherlands (6)	Netherlands (6)
	Estonia (7)	Estonia (7)	Estonia (7)
	Finland (8)	Finland (8)	Finland (8)
	Spain (9)	Spain (9)	Spain (9)
	Germany (10)	Germany (10)	Germany (10)
*Bottom 10 countries*	Tunisia (50)	Tunisia (50)	Tunisia (53)
	Malaysia (51)	Malaysia (51)	Malaysia (54)
	New Zealand (52)	India (52)	India (55)
	India (53)	New Zealand (53)	New Zealand (56)
	Jordan (54)	Jordan (54)	Jordan (57)
	Indonesia (55)	Indonesia (55)	Indonesia (58)
	Myanmar (56)	Saudi Arabia (56)	China, People’s Rep. (59)
	Saudi Arabia (57)	Myanmar (57)	Myanmar (60)
	China, People’s Rep. (58)	China, People’s Rep. (58)	Saudi Arabia (61)
	Philippines (59)	Philippines (59)	Philippines (62)

*Note*: While the FDI Index does not rank countries the numerical scores produce a hierarchy of performance.

*Source*: Data retrieved from OECD website, available at: http://stats.oecd.org/Index.aspx?datasetcode=FDIINDEX#

Like the EDB ranking, the FDI Index purports to measure objective national performance but is based on contested assumptions about the benefits of open markets and the pathologies of economic regulation. As investment regulations that do not grant foreign capital the same market access conditions enjoyed by domestic firms are framed negatively as restrictions, the FDI Index represents a national economy that is open to international investment flows as the ideal. The average score across the 35 OECD member states in 2016 was only 0.067, indicating an almost complete absence of restrictions on FDI. Rising economic powers scored significantly higher, indicating a greater reliance on controls to restrict such inflows; for example, Brazil scored 0.101, Russia 0.187 and India 0.212. China received one of the highest scores (signalling greater restrictiveness) of the 62 countries rated in 2016, with 0.327 (OECD, 2017). As market analysts have noted, however, there is no automatic correlation between the restrictiveness of countries’ investment rules and actual FDI inflows (Garry, 2013). The case of China provides an apt illustration here. Despite receiving the second worst score in the 2014 FDI Index, in the same year, the country reached the status of being ‘the world’s largest recipient of FDI’ (UNCTAD, 2015: ix).

Furthermore, the FDI Index cannot capture differences in the implementation and enforcement of statutory restrictions across countries, nor does it measure ‘the nature of corporate governance, the extent of state ownership, and institutional or informal restrictions’, or sub-national policies (Kalinova et al., 2010: 6, 9). Thus, the FDI Index merely constructs an image of countries’ openness to international economic integration and gauges how close their policies come to the OECD’s ideal type of an open economy; it is not a measure of national success in attracting foreign investment or of the degree of international economic integration *per se*.

The classification and coverage of different economic sectors in the FDI Index promotes an image of the ideal economy as one that is almost entirely free from statutory restrictions on FDI. To perform well in the FDI Index, countries need to have designed a national regulatory framework according to this image, with negative implications for countries that may prefer policy alternatives. In a fashion similar to the EDB ranking, the FDI Index privileges an image of business freedom — in this case, the equitable treatment of foreign investors and domestic businesses — which encourages countries to gradually converge towards the OECD member state average at the very bottom of the 0–1 scale of FDI openness, based on the principle of ‘progressive liberalization’ (Williams, 2008: 125). For the purposes of composing the FDI Index, any policy measure that discriminates between foreign and domestic investors counts as a restriction (Kalinova et al., 2010: 6). Policy alternatives to a liberalized foreign investment regime, which may offer important benefits for a country’s economic performance and development trajectory, are classified as aberrations from the norm. The implicit assumption promoted through the IO benchmark is that such policies harm economic growth and development, which results in bad scores.

Overall, then, the FDI Index rests on a division between ‘good’ and ‘bad’ foreign investment rules. This analytical approach contributes to the maintenance of a transnational policy paradigm that is centred on the assumed benefits of unrestricted capital mobility for economic growth. Its purpose is to identify statutory barriers to investment across a wide range of policy areas encompassing primary (agriculture), secondary (manufacturing) and tertiary (services) industries, as well as rules on the acquisition of land and real estate investment. This binary conception of countries’ rules for governing foreign investment supplements the OECD’s general promotion, not least through the 1989 amendment to its Code of Liberalization of Capital Movements, of capital mobility for both long-term and short-term investments (Abdelal, 2006: 14). In this image of the world, unfettered international capital flows offer host countries only economic benefits and no costs. Among other things, this image obscures the potential for transnational corporations to shift taxable revenue offshore through transfer-pricing practices within ‘global wealth chains’ (Seabrooke and Wigan, 2017). The benchmark thus sidelines the possibility that the equitable treatment of foreign and domestic investors might instead grant the former a structural advantage over the latter on what is already an uneven playing field.

Although references to the FDI Index are common in policy conversations, as well as in behind-the-scenes negotiations, it does not produce many news headlines or stir much controversy. Instead of high public visibility, the indirect power of the FDI Index lies in its widespread use by IOs and governance forums. Importantly, a range of additional OECD surveillance instruments make extensive use of the FDI Index, which extends the reach of both the benchmark and the organisation itself because it helps to disseminate OECD concepts and standards of ‘best practice’. For example, the FDI Index forms one element of the OECD Indicators of Product Market Regulation (PMR) (Kalinova et al., 2010: 5). The PMR indicators provide international comparative measures of economy-wide policy regimes that promote or inhibit market competition in 34 OECD and 22 non-OECD countries (the FDI Index is used to identify barriers to investment). The PMR, in turn, shapes the definition of policy priorities in the flagship OECD annual publication *Going for Growth* (first published in 2005), which compares structural policy developments across OECD members, identifies desirable economic reforms and assesses national progress towards the adoption of recommended policy reforms from year to year. The *Going for Growth* report also forms the basis of the OECD’s multilateral assessment role within G20 working groups. Specifically, it informs the OECD’s input to the *G20 Framework for Strong, Sustainable and Balanced Growth* as well as the *G20 National Growth Strategies*, which were designed as a multilateral mechanism to pursue the goal of expanding economic growth by 2% by 2018 through growth-enhancing reforms and the monitoring of countries’ progress in achieving them (Schwanen, 2010).

**Figure 3.**
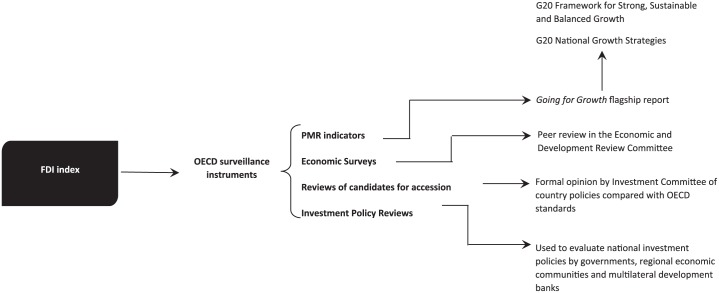
How the OECD’s FDI Index is used in transnational governance processes.

In addition to the PMR indicators, the FDI Index is used in OECD Economic Surveys, which are conducted every two years for each OECD member state and several non-OECD countries. It also feeds into countries’ ‘roadmaps’ for accession to the OECD, where the OECD Investment Committee prepares a formal opinion on a candidate country’s investment policies, measured against OECD ‘best practice’ standards, which informs the negotiation of policy reforms as part of the accession process. Finally, the FDI Index forms an important component of OECD Investment Policy Reviews, which the organization undertakes in response to an official request to evaluate a country’s investment trends and policies. Multi-agency government task forces, regional economic communities such as the Association of Southeast Asian Nations (ASEAN), the Southern African Development Community (SADC), and the New Partnership for Africa’s Development (NEPAD), and the World Bank Group and other multilateral development banks have used Investment Policy Reviews to shape policy reform initiatives and to signal a government’s desire to improve the investment climate. Figure 3 depicts a simplified illustration of the convoluted process whereby the transnational knowledge created by the FDI Index is disseminated across multiple surveillance instruments and governance forums.

Like the EDB ranking, the OECD’s FDI Index is part of a neoliberal transnational policy paradigm that extols the virtues of opening up the economy and reducing the regulatory role of the state over business activities. While global governance actors have promoted this broader agenda in a variety of ways (Abdelal, 2006; Ban, 2016; Kentikelenis et al., 2016), IO benchmarking practices play specific roles in disseminating and reinforcing global policy norms by representing contested images of the world in simplified form as expert knowledge. The FDI Index makes foreign investment regulations visible as a policy problem and thus amenable to standardized reforms across different national contexts. It serves to bolster the OECD’s wider efforts to advocate for the removal of foreign investment restrictions via the production of comparative international metrics that track deviations from the norm. When domestic policymakers try to convince a sceptical public that liberalization is not just wise, but inevitable, because national competitiveness is at stake, they are invoking a particular conception of the economy as the ideal. The FDI Index thus presents and promotes a distorted image of the world by labelling regulations that *might* restrict investment as a policy problem — regardless of whether they actually restrict FDI flows or how they interact with a government’s other social and economic objectives.

## IO benchmarking as paradigm maintenance

IO benchmarks make us view and engage with the world on particular terms by influencing what problems we see in a given policy domain and how we look at them to craft political solutions. Benchmarks can therefore misrepresent an issue as a result of construct or content validity problems. Moreover, as the investigation of these two cases has demonstrated, IO benchmarks sanction existing hierarchies in world politics by framing contested choices of problem definition and conceptualization as based upon the neutral application of rational-scientific expertise. In both cases, the ratings or rankings that are obtained in the respective issue areas are presented as objective measures of country performance; the benchmarks clearly assign responsibility for success and failure to national authorities, rather than to broader socio-political structures and dynamics. Furthermore, while the limited number of countries ranked at the top and bottom take the limelight, there is little mobility at either end of the comparative performance scales produced and promoted by IOs: the best- and worst-performing countries in these two benchmarks have remained fairly constant over time (see Tables 2 and 3).

By holding up some states as role models to emulate while framing others as underperformers who have to change, IO benchmarks promote images of the world as divided into cases of ‘success’ and ‘failure’. By linking the attribution of praise and blame to knowledge-based practices, IO benchmarking specifies ‘what is normal and desirable’ — and, by implication, ‘what is abnormal and undesirable behavior’ (Towns, 2012: 180). The articulation of superior and inferior qualities in IO benchmarks serves as a mechanism for (re)producing social hierarchies among states. At the same time, it locates the causal factors for country performance at the domestic level, thereby marginalizing sources of structural power that might be equally responsible for the benchmarked outcomes.

Our empirical illustrations highlight that IO benchmarks focus attention on a limited set of data input factors. The simplistic numerical rankings that this process yields risk narrowing the scope of political debate down to one-size-fits-all checklists of the technical adequacy of institutional design, with ticks and crosses (as in the case of the EDB ranking) to measure the degree of conformity with what an IO promotes as global ‘best practice’. As the preceding discussion shows, the promotion of a specific interpretation of highly political concepts through the use of what is perceived as value-neutral factual evidence reinforces a set of particularistic values as broadly accepted standards.

IO benchmarking also shapes the ways in which we assign meaning to contested and value-laden concepts such as investment restrictions and business regulation. Specifically, IO benchmarking practices influence processes of sensemaking by either challenging or reinforcing prevalent normative conceptions of problem definition and solution. How an issue is articulated and operationalized in IO benchmarks fosters the development of a normative consensus on the appropriate scope and targets of political action consistent with the agenda and mandate of the organization producing them (Charnysh et al., 2015: 327–328). The ability of IO benchmarks to shape how actors think about an issue is also closely connected to their capacity to influence transnational processes of policy diffusion, whereby knowledge about governance and policy fads travels around the world across political settings (Stone, 2004). The available transnational supply of policy lessons influences how actors in a specific political domain devise strategic interventions in an issue area and what kinds of policy changes are prescribed or discouraged. Global benchmarks amplify and reinforce these diffusion mechanisms while limiting the supply of alternative policy lessons.

A number of scholars have recently noted a contemporary shift within the global development regime away from the transnational paradigm that characterized the ‘Washington Consensus’ era of the 1980s and 1990s towards a greater focus on ‘best practices’ and ‘measurable results’ (Babb and Chorev, 2016: 94–97; see also Best, 2017; Kentikelenis and Seabrooke, 2017). Notwithstanding the importance of these changes, our analysis suggests that IO benchmarking techniques enable the core precepts of this transnational paradigm to be (re)deployed as quantitative metrics of development progress. Indeed, many of the controversial free market ideas associated with the ‘Washington Consensus’ paradigm continue to be championed both directly and indirectly through IO benchmarks. Largely because of the processes of simplification and extrapolation that are required to produce comparable aggregate indicators that can be expressed numerically (Broome and Quirk, 2015a: 827), IO benchmarking remains oriented towards a top-down grand vision of development and economic governance even as the same organizations may embrace a more decentralized approach in their other activities. In the two cases of IO benchmarking that we have examined here, therefore, the EDB ranking and the FDI Index serve as mechanisms of paradigm maintenance by other means. Such benchmarks increase the staying power of existing paradigms by obscuring specific policy positions within the application of organizational expertise. In this respect, the EDB ranking and the FDI Index are pertinent examples of a wider global governance trend towards the cloaking of contested political ideas and normative agendas in the more legitimate language of objective performance measurement, numerical indicators and peer comparisons.

Overall, our analysis of both cases suggests that the promotion of contested images of the world as value-neutral expert knowledge can have far-reaching ramifications when these function to focus political attention and to attribute responsibility for outcomes. Not only do benchmarks construct images of the world that orient attention towards certain dimensions of an issue while obscuring others, they also direct towards national governments praise for the ‘good’ and blame for the ‘bad’ outcomes showcased by a benchmark. When they marginalize or obscure salient factors, or when they misrepresent the degree to which national authorities are responsible for performance in a given area, benchmarks can significantly distort political debates and policymaking processes. The indirect power of IO benchmarking is therefore rooted in its capacity to influence what counts as legitimate knowledge, what issues occupy political debates, how to think about those issues, what policies are advocated and who is assigned responsibility for successes and failures. In short, IO benchmarking contributes to setting the boundaries of ‘political possibility’ (Holland, 2011) by narrowing the space available for political contestation and magnifying the social pressures on states to conform to transnational policy paradigms.

## Conclusion

The role that IOs play as evaluators of national policy designs, economic performance and social outcomes matters. This article has shown that global benchmarking practices by IOs create prisms that shape the interpretation of national performance in world politics. However, although IOs draw upon their expert status to do so, the benchmarks that they produce should not be understood as robust and transparent registers of success and failure. Rather, our analysis suggests that with these prisms, we see distorted images of how different countries compare in terms of their national performance on different political issues within the limitations of a specific transnational policy paradigm. In the two cases examined in this article — the World Bank–IFC EDB ranking and the OECD FDI Index — the principles underlying the construction of each benchmark are based on pro-market (minimal regulation) assumptions about how economic governance ought to work.

For IOs, however, benchmarking is highly appealing. Due to the indirect power of comparative performance metrics, benchmarks can augment other avenues through which IOs may exercise both direct and indirect power in world politics, such as the application of material incentives and loan conditionality or processes of socialization and stigmatization. At the same time, it can increase the traction of an IO’s broader efforts to shift the parameters within which national elites undertake deliberations, enter negotiations, make decisions, formulate goals and order priorities. Perhaps most significantly, benchmarking expands the scope of the power of an IO to classify complex political, economic and social phenomena, as well as to provide an evidential basis for labelling some types of states and governance techniques as ‘best practice’ while delegitimizing others. IO benchmarks are thus integral to understanding the complex ways in which policy norms and standards that reflect a particular transnational paradigm are both legitimated and diffused across political settings.

The (re)production of social hierarchies between states via transnational knowledge practices that establish ideal and pathological models of state action is underpinned by an appeal to the authority of rational-scientific expertise housed within IOs. While global benchmarking practices are often used by civil society organizations to challenge existing policy paradigms in world politics (Seabrooke and Wigan, 2015), the use of benchmarks by IOs to rate and rank national performance in the two cases examined here serves to maintain a transnational paradigm that is centred on extolling the benefits of open markets and is critical of the regulatory role of the state in governing economic activity. We have illustrated that benchmarking practices by IOs both configure reputational incentives for national policymakers to achieve a better score in global ratings and rankings, and encapsulate appraisals of national performance within a problematic logic of comparison. Yet, national performance in a given issue area is not independent, but, at least in part, contingent upon diverging contemporary structural conditions and historical legacies of domination. Notions of national success and failure are thus far more relative concepts than glossy country rankings imply.

This article has three main implications for future research on the role of IOs as actors that exercise expert authority in world politics. First, it points to the importance of further investigating the complex linkages that connect different modes of transnational knowledge production with efforts to challenge or maintain dominant paradigms across various types of global governance actors. In particular, more research is needed to examine how these linkages operate through mechanisms of transnational socialization and stigmatization, as well as through transnational evaluation. This agenda for future research includes exploring how the production of global performance metrics in one field might influence knowledge practices in others, as well as how transnational knowledge is recursively deployed across different political settings, and with what effects. Second, future research will need to specify the scope conditions under which the knowledge practices of IOs enable them to legitimize claims to issue expertise, including how the indirect power of benchmarking interconnects with other forms of direct and indirect power. Our discussion in the final section has outlined some of the links that larger empirical studies could investigate to gain additional insights into the interconnections of power, knowledge and expertise in global governance. Finally, our research suggests that scholars themselves must approach global benchmarks with a more critical and sceptical stance on the legitimacy of using comparative metrics to construct evidence about comparative national performance or to track trends in a particular issue area. Reliance on these problematic tools to construct transnational knowledge distorts how we interpret the world, as well as how we seek to change it.

## Notes

1. The EDB ranking is a flagship report of the World Bank Group, which comprises five IOs. The IFC produces the EDB Report in collaboration with the International Bank for Reconstruction and Development and the International Development Association (hereafter ‘the World Bank’).

2. *N* = 275 in version 1.9 as of 5 July 2017. Available at: http://warwick.ac.uk/globalbenchmarking/database
